# Effect of exercise on chemically-induced colitis in adiponectin deficient mice

**DOI:** 10.1186/1476-9255-9-30

**Published:** 2012-08-21

**Authors:** Arpit Saxena, Emma Fletcher, Bianca Larsen, Manjeshwar Shrinath Baliga, J Larry Durstine, Raja Fayad

**Affiliations:** 1 Exercise Science, Applied Physiology Division, University of South Carolina, Columbia, SC, USA; 2 Father Muller Medical College, Kankanady, Mangalore, Karnataka, India; 3 University of South Carolina, Arnold School of Public Health, 921 Assembly St. room 301, Columbia, SC, 29208, USA

**Keywords:** Adipokines, Cytokines, Inflammation, Epithelial cell proliferation, Intestine

## Abstract

**Background:**

Inflammatory bowel diseases are associated with increased adiponectin (APN) levels, which may exert pro-inflammatory effects in these individuals. Since habitual exercise may increase APN, the aim of this study was to determine how exercise training affects mice with acute colitis.

**Methods:**

Male adiponectin knock out (APNKO) and wild type (WT) mice (C57BL/6) were randomly assigned to 4 different groups: 1) Sedentary (SED); 2) Exercise trained (ET); 3) Sedentary with dextran sodium sulfate (DSS) treatment (SED + DSS); and 4) Exercise trained with DSS (ET + DSS). Exercise-trained mice ran at 18 m/min for 60 min, 5d/wk for 4 weeks. Subsequently, the ET + DSS and the SED + DSS mice received 2% DSS in their drinking water for 5 days (d), followed by 5d of regular water.

**Results:**

The clinical symptoms of acute colitis (diarrhea, stool haemoccult, and weight loss) were unaffected by exercise and there was no difference between the APNKO and WT mice (p > 0.05) except on day 39. However, the clinical symptoms of the DSS-treated APNKO mice were worse than WT mice treated with DSS and had increased susceptibility to intestinal inflammation due to increased local STAT3 activation, higher IL-6, TNF-α, IL-1β and IL-10 levels, and as a result had increased intestinal epithelial cell proliferation (p < 0.05). Exercise training significantly decreased pro-inflammatory cytokines including IL-6, TNF-α and IL-1β (p < 0.05) in the DSS + EX APNKO mice but had no effect on epithelial cell proliferation. Exercise was also found to significantly decrease the phosphorylation expression of STAT3 in both WT and APNKO mice in DSS + EX group when compared to DSS + SED.

**Conclusions:**

Exercise training may contribute in alleviating the symptoms of acute colitis and APN deficiency may exacerbate the intestinal inflammation in DSS-induced colitis.

## Background

Inflammatory Bowel Disease (IBD), comprised primarily of Crohn’s disease (CD) and ulcerative colitis (UC), are chronic inflammatory disorders of the gastrointestinal tract that typically results in significant morbidity due to high incidence of diarrhea, abdominal pain, rectal bleeding and malnutrition [1,2]. Each year IBD accounts for approximately 700,000 physician visits and 100,000 hospitalizations in the U.S. alone [1] and affects approximately 3.4 million people across the United States and Europe [2]. Despite being idiopathic disorders, the current leading hypothesis for the pathogenesis of IBD is that the interacting environmental and genetic factors, in combination with alterations in the microbial intestinal flora, trigger intestinal barrier disruption leading to an enhanced or aberrant immunologic responsiveness to the commensal bacteria resident in the gut lumen [2-4]. Typically, the immune system of the gut exists in a state of homeostasis, however, with IBD; immune regulation is altered in such a way that there is a disturbed balance between both pro- and anti- inflammatory mechanisms, causing the intestinal immune system to remain chronically activated, and thus inflamed [1,3-5]. Several pro-inflammatory cytokines such as IL-1β, IL-6 and TNF-α produced by T cells, macrophages, epithelial and mesenchymal cells play a key role in the modulation of the intestinal immune system to induce, amplify and perpetuate inflammation in these individuals [4].

Recently, the adipokine adiponectin (APN) has been implicated in the etiology and severity of IBD [6-8]. APN, a long polypeptide derived from adipose tissue, is typically found in abundant concentrations within the peripheral circulation; however, its levels are reduced with insulin resistance, type 2 diabetes and obesity [9-11]. APN is structurally homologous to collagen VIII, X, complement factor C1q [12] and the TNF family [13], and therefore plays an important role in inflammation and the immune system [10] through the regulation of both cytokine production and their resultant systemic effects [14-16]. APN is typically viewed as an anti-inflammatory agent that; inhibits the Nuclear Factor-kappa B (NFκB) signal transduction pathway, thus contributing to the inhibition of TNF-α and the attenuation of IL-6, and APN concomitantly increases the expression of the anti-inflammatory cytokines IL-10 and IL-1 receptor antagonist (IL-1Ra) [14,17]. Furthermore, reduced levels of APN are inversely correlated with the incidence of atherosclerosis and insulin resistance [9,11]. Conversely, in chronic inflammatory or autoimmune diseases such as IBD and arthritis, where there is a tendency towards a negative energy balance, APN may contribute a paradoxical pro-inflammatory effect through the induction of pro-inflammatory cytokine and chemokine production such as IL-6, IL-1β, TNF-α, IL-8, granulocyte-macrophage colony stimulating factor, and monocyte chemotactic protein-1 [6-8]. An upregulation of the signal transducers and activators of transcription (STAT)3 signaling pathway may account for some of the pro-inflammatory actions of APN in IBD but this requires further confirmation. In addition, APN binds to and inhibits certain protective growth factors such as heparin binding epidermal growth factor (HB-EGF) and basic fibroblast growth factor (bFGF), necessary for maintaining colonic epithelial cell integrity and proliferation, and regulating adhesion molecule expression and NFκB activation [6-8].

There is substantial evidence that regular physical activity has profound implications on overall wellbeing as a result of complex interactions between interrelated physiological mechanisms [18-20]. In many clinical and non-clinical populations, habitual endurance exercise elicits anti-inflammatory effects through the reduction of the pro-inflammatory cytokines TNF-α, interferon-γ, IL-1β and IL-6, the acute phase protein C-reactive protein, the pro-inflammatory adipokines leptin and ghrelin and free radicals, as well as increasing the levels of the anti-inflammatory cytokines IL-10 and IL-1Ra [21-23]. Although controversial, the general consensus is that endurance training will also increase the expression and circulation of APN and its receptors in obese and diabetic patients and plays an anti-inflammatory role in these individuals [24-26]. However, should APN increase with exercise training in patients with IBD, then exercise may further augment the inflammatory state associated with IBD, or alternatively prevent the anti-inflammatory effects of exercise training. Evidence does exist that low intensity exercise training is in fact protective against oxidative colonic damage in wild type (WT) rats with dextran sodium sulfate (DSS)-induced colitis [27], however, given that the greatest overall benefits of exercise training observed in both the general population and in other chronic conditions occur when the least active individuals become moderately active [20], it is unknown whether current moderate intensity exercise recommendations would be beneficial or harmful to individuals with IBD. Furthermore, it is unknown whether the presence of APN will impede the benefits typically associated with exercise training. Therefore, the aims of this study were to determine the effect of four weeks of moderate-intensity treadmill running on 1) the clinical and histopathological symptoms associated with acute colitis, 2) intestinal levels of the cytokines IL-1β, IL-6, TNF-α and IL-10 and serum adiponectin, 3) intestinal STAT3 and phosphorylated (p)-STAT3 expression, and 4) colonic epithelial cell proliferation in APN deficient mice recovering from chemically-induced acute colitis.

## Methods

### Animals

Four week old male APN knock out (APNKO) (n = 16) (bred in the animal facility at the University of South Carolina (USC)) and male WT mice (C57BL/6) (purchased from Jackson’s Laboratory) (n = 16) were housed and treated in the animal facility at USC. The mice were on a 12:12 h light–dark cycle in a low stress environment (22°C, 50% humidity and low noise) and had access to food (Purina Chow) and water ad libitum. All exercise trials were performed at the end of the dark cycle (0700). All animal care followed institutional guidelines under a protocol approved by the Institutional Animal Care and Use Committee at the University of South Carolina, which were in adherence with the animal care standards set by the Scandinavian Journal of Medicine and Science in Sports. All the experiments were repeated twice (n = 4).

### Experimental groups

APNKO and WT mice were randomly assigned to 4 different groups of equal number (n = 4 mice per group): 1) Sedentary (C + SED); 2) Exercise trained (C + EX); 3) Sedentary with 1 cycle of DSS treatment (DSS + SED) (to induce acute colitis); and 4) Exercise trained with 1 cycle of DSS (DSS + EX).

### Exercise training protocol

Prior to the initiation of exercise training, the mice assigned to the treadmill running groups were acclimated to the treadmill for 3 days. Acclimation entailed running at the end of their dark cycle (0700) at gradually increasing speeds (10, 12, 16, and 18 m/min) and a 5% incline for 20 min [28]. Following the acclimation period, the mice were trained utilizing a treadmill running program. The running protocol consisted of a 5 min warm-up (12 m/min), followed by 55 min at 18 m/min and 5% incline. The mice ran 5d/wk at the end of their dark cycle (0700) for a total of 4 weeks. The mice were encouraged to run by a gentle tap on the tail or hindquarters - no electrical shock or other method was used. Control mice were kept in their respective cages next to the treadmill during all exercise and acclimation sessions.

### Induction of acute colitis

Following the completion of the 4 week exercise training program, colonic inflammation was induced in the WT and APNKO mice assigned to the DSS + SED and DSS + EX treatment groups. These mice received 2% DSS (MP Biochemicals, MW 36,000 - 50,000) dissolved in their drinking water for 5 days, followed by 5 days of regular drinking water. Control mice had access to regular drinking water without DSS for the duration of the study. All of the exercise trained mice (i.e., the C + EX and the DSS + EX groups) continued to follow the moderate intensity treadmill running protocol for the duration of DSS treatment.

### Monitoring animal health

During and following DSS treatment, individual mice were observed daily in a blinded fashion for clinical signs of disease manifested by weight loss, positive fecal haemoccult, and diarrhea [7]. Weight loss was ranked by assigning points as follows: 0 = 0–5% weight loss; 1 = 6–10% weight loss; 2 = 11–15% weight loss; 3 = 16–20% weight loss; and 4 = >20% weight loss. The appearance of diarrhea was scored as 0 = well-formed pellets, 2 = pasty and semi-formed stools that do not adhere to the anus, 4 = liquid stools that adhere to the anus. Appearance of blood in the stools was scored as 0 = no blood, 2 = positive haemoccult, 4 = gross bleeding using a haemoccult kit (Beckman Coulter). The clinical score was then determined by totaling the weight loss, haemoccult, and diarrhea scores.

### Tissue collection

At the end of the study (i.e., day 43, 24 hr after the last exercise session) the animals were injected i.p. (1 mg/mouse) with Bromodeoxyuridine (BrdU) (Sigma Aldrich). The mice were euthanized 2 hr later by cervical dislocation and tissue collection was performed as a non-survival surgery. The colon was removed from the distal end of the cecum to the anus, flushed with PBS (EMD Chemicals) and two 2 mm sections of the transverse colon were cut. One section was opened longitudinally, placed in 24-well plates containing 1000 μl of RPMI 1640 medium supplemented with 1% penicillin-streptomycin (Mediatech, Inc) per well and incubated for 12 hours in a NUAIRE incubator at 37°C and 5% CO_2_. The cytokine-conditioned RPMI medium was then centrifuged in eppendorf tubes at 10,000 g for 10 min at 4°C and the supernatant was collected and stored at ≤ −20°C until use. The second 2 mm section of the transverse colon was placed whole in a tissue collection cassette, fixed in formalin (Fisher Scientific) and stored in 70% ethanol, which was then cut in to 5–6 μm thin sections for BrdU and Hematoxylin and Eosin staining. A standard protocol for H&E staining was used. Quantification of severity of colitis was estimated on the scale of 4, where 0 = no infiltration and inflammation; 2 = moderate infiltration and inflammation; and 4 = severe inflammation with distorted crypts and infiltration of immune cells. All the images were taken in 20× magnification with Nikon e600 microscope. The scale bar represents 120 μm.

### Measuring intestinal cytokine and serum adiponectin concentrations

The cytokine-conditioned RPMI medium was used to quantify local concentrations of IL-6, IL1-β, TNF-α and IL-10 (BD Biosystems) [7,29] while mice serum was used to calculate the concentration of adiponectin using commercially available immuno-assay ELISA kits, according to the manufacturer’s instructions. Local cytokine concentrations were normalized by protein content contained in the colon supernatant as determined via a Bradford protein assay.

### STAT3 and pSTAT3 expression

STAT3 and pSTAT3 expression (Cell Signaling Technology) was analyzed by Western blot and normalized relative to GAPDH expression (R & D Systems) using equal amounts of proteins (12 μg/mL).

### Estimating epithelial cell proliferation

Following tissue sectioning, the previously fixed sections of the transverse colon were stained for BrdU using a BrdU in-situ detection kit (BD Pharmingen). The number of BrdU positive cells was then counted in 10 well-defined crypts for each animal using light microscopy.

### Statistical analysis

XLStat for Windows (version 2009.4.07) statistical software was used to perform the statistical analysis. Statistical significance was accepted at the p < 0.05 level of confidence. Analysis of variance (ANOVA) and post-hoc tests (using Fisher (LSD) correction) were used to compare the clinical symptoms, IL-6, IL-1β, TNF-α and IL-10 concentrations, the relative intensity of STAT3 and pSTAT3 expression, and the amount of colonic epithelial cell proliferation between the different experimental groups.

## Results

### Moderate intensity endurance exercise training does not alter exercise behavior or the clinical symptoms associated with acute colitis

**Figure 1:**
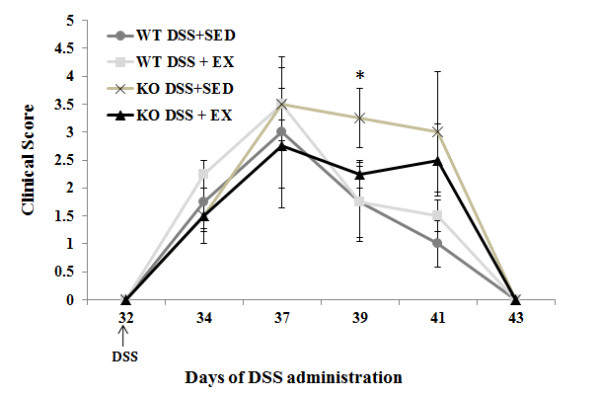
**Clinical score.** Clinical score has been plotted against the days of DSS administration. Two-way repeated measure analysis of variance (ANOVA) was used to calculate significant difference between various groups. “*” represent p < 0.05 between APNKO mice in DSS + EX and DSS + SED group.

In order to study the effect of exercise training and APN deficiency on the clinical symptoms associated with acute colitis, APNKO and WT mice with and without DSS treatment underwent a four weeks moderate-intensity treadmill running program. No changes in exercise behavior were observed in any of the DSS-treated animals, who were able to maintain the prescribed training protocol for the duration of the study. Furthermore, the clinical symptoms were unaffected by exercise (p > 0.05) in the DSS + EX WT mice when compared to their sedentary counterparts. In the DSS + EX group, APNKO mice showed significant decrease in the clinical score (p < 0.05) at day 39 when compared to DSS + SED (Figure 1). However, sedentary APN KO mice treated with DSS had worse clinical manifestations including greater score than WT treated with DSS. There was additionally no significant difference in clinical symptoms between the untreated APNKO and WT mice (p > 0.05) (Figure 1).

### Exercise reduced histopathological score in APNKO mice treated with DSS

**Figure 2:**
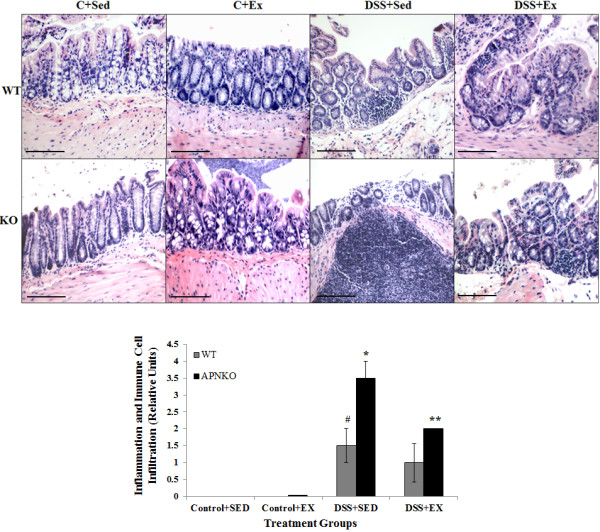
**Inflammation and immune cell infiltration.** (A) Representative Hematoxylin and Eosin stained images of the transverse colon section for all the treatment groups. (B) Graph shows quantitative measure of the degree of inflammation and immune cell infiltration in the transverse colon sections of both control and DSS treated groups. One-way ANOVA was used to calculate the significant difference. # vs * p < 0.04 and * vs ** p < 0.03.

Hematoxylin and Eosin staining was performed on the transverse colon sections to investigate the degree of inflammation and immune cell infiltration in all the treatments groups (Figure 2A). APNKO mice in DSS + SED group showed significantly higher score than its counterpart in DSS + EX group and with WT mice in the same group (p < 0.04). No significance was found in between the WT mice in DSS + EX and DSS + SED group and between APNKO and WT mice in DSS + EX group (Figure 2A and B).

### DSS-induced acute colitis elevates intestinal cytokine production

**Figure 3:**
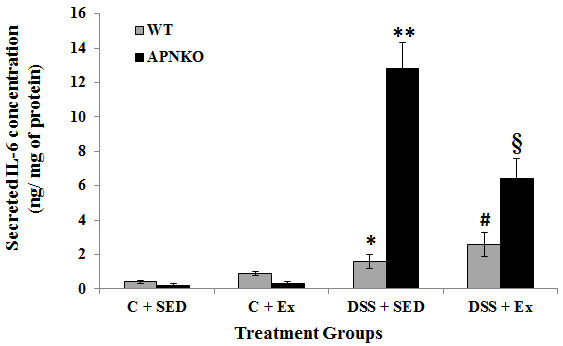
**Effect of exercise training on intestinal IL-6 production within transverse colon tissue.** Secreted levels of IL-6 in colon culture supernatants of sedentary and exercise-trained WT and APNKO mice with and without DSS-induced colitis. Two way ANOVA was used to calculate significant difference between various groups. (APNKO) DSS vs Control, ** vs C + SED APNKO p < 0.001, ** vs *, p < 0.001, (WT) DSS vs Control p < 0.02, § vs # p < 0.05, ** vs § p < 0.03. Data are means ± SE. N = 4 mice per group.

**Figure 4:**
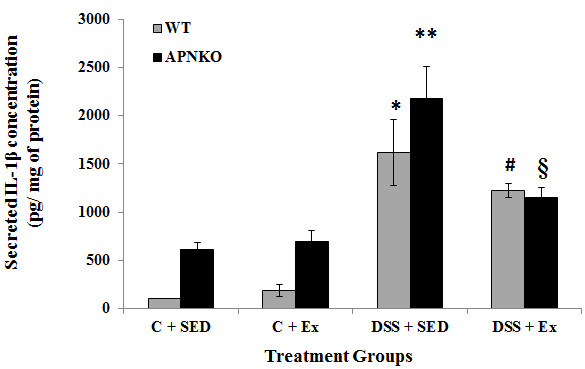
**Effect of exercise training on intestinal IL-1β production within transverse colon tissue.** Secreted levels of IL-1β in colon culture supernatants of sedentary and exercise-trained WT and APNKO mice with and without DSS-induced colitis. Two way ANOVA was used to calculate significant difference between various groups. (APNKO) DSS vs Control p < 0.01, (WT) DSS vs Control p < 0.002, ** vs C + SED APNKO p < 0.01, ** vs § p < 0.05, C + SED APNKO vs WT, C + EX APNKO vs WT p < 0.05. Data are means ± SE. N = 4 mice per group.

**Figure 5:**
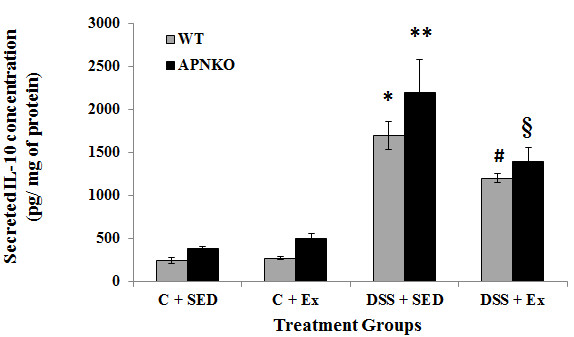
**Effect of exercise training on intestinal IL-10 production within transverse colon tissue.** Secreted levels of IL-10 in colon culture supernatants of sedentary and exercise-trained WT and APNKO mice with and without DSS-induced colitis. Two way ANOVA was used to calculate significant difference between various groups. (APNKO) DSS vs Control p < 0.01, (WT) DSS vs Control p < 0.003, ** vs C + SED APNKO p < 0.002. Data are means ± SE. N = 4 mice per group.

**Figure 6:**
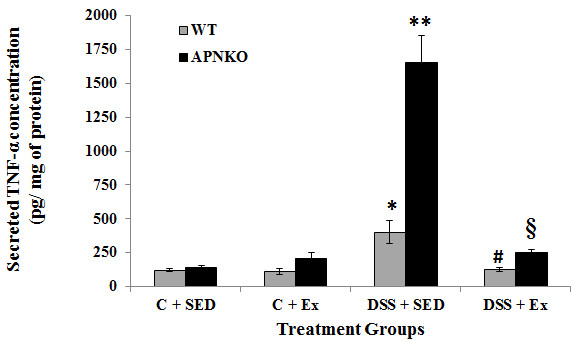
**Effect of exercise training on intestinal TNF-α production within transverse colon tissue.** Secreted levels of TNF-α in colon culture supernatants of sedentary and exercise-trained WT and APNKO mice with and without DSS-induced colitis. Two-way ANOVA was used to calculate significant difference between various groups. (APNKO) DSS vs Control p < 0.03, ** vs C + SED APNKO p < 0.001, ** vs * p < 0.001, § vs # p < 0.05, ** vs § p < 0.004. Data are means ± SE. N = 4 mice per group.

Secreted intestinal cytokine concentrations were determined in the transverse colon section of APNKO and WT mice treated with DSS and control group with or without exercise intervention DSS-treated mice showed significantly higher secretion of IL-6, IL-1β, TNF-α and IL-10 when compared to the control group or no treatment (p < 0.03) (Figures 3, 4, 5 and 6). The higher degree of significance were primarily due to the significant difference between the DSS treated and control group in APNKO mice for IL-1β (p < 0.01), IL-6 (p < 0.001), TNF-α (p < 0.03) and IL-10 (p < 0.001) and WT mice for IL-1β (p < 0.002), IL-6 (p < 0.02) and IL-10 (p < 0.001). No significant difference in the secreted levels of TNF-α was found between the DSS treated and control WT mice. Further significance could be found in the APNKO mice in DSS + SED when compared with Control + SED (p < 0.01).

### APN deficiency is associated with greater pro-inflammatory cytokine production

APNKO mice showed significantly higher secretion of IL-6 (p < 0.001) and TNF-α (p < 0.001) in DSS + SED group when compared to WT mice (Figures 3 and 6). However, no significance was obtained for either IL-1β or IL-10. Similar results were obtained with the APNKO mice in DSS + EX group with significantly higher secretion of IL-6 (p < 0.05) and TNF-α (p < 0.05) when compared to WT mice and no significance for either IL-1β or Il-10 was obtained. In Control groups, significantly higher secretion of IL-1β was observed in the APNKO mice when compared to the wild counterpart (p < 0.05).

### Moderate intensity exercise training decreases pro-inflammatory cytokines in the colon of APNKO mice with acute colitis

To determine the effect of habitual endurance training on the secreted intestinal pro-inflammatory cytokines, both APNKO and WT mice were run on a motorized treadmill at a moderate intensity 5d/wk for 4 weeks. Exercise training was found to have significant effect on the secretion of IL-6, IL-1β, TNF-α and IL-10 from some of the WT and APNKO mice in DSS treated and control group. APNKO mice in DSS + EX group showed significantly lower secretion of IL-6 (p < 0.03), IL-1β (p < 0.05) and TNF-α (p < 0.004) when compared to APNKO mice in DSS + SED group. However, DSS treated WT mice with sedentary behavior showed no significant change in the secretion of IL-6, IL-1β, TNF-α and IL-10 when compared with mice given exercise as an intervention. Control + EX group showed higher secretion of IL-6 and TNF-α in WT and APNKO mice respectively when compared to Control + SED, but the levels of other cytokines were not found to be significantly altered. In DSS treated APNKO and WT mice slightly but not significant decrease in the secretion of IL-10 was observed in the trained mice as compared to sedentary group.

### Decrease in the serum adiponectin level with in DSS induced colitis with concomitant increase in moderate intensity exercise in WT mice

**Figure 7:**
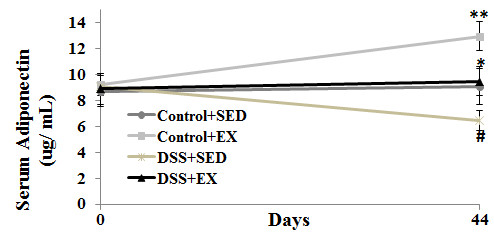
**Serum adiponectin in WT mice.** The graph represents the serum APN in WT mice four group. Two-way ANOVA was used to calculate significant difference between various groups. * vs ** p < 0.03, * vs # p < 0.05, control + SED vs ** p < 0.01, control + SED vs # p < 0.05.

To study the effect of DSS induced colitis and moderate intensity exercise on serum APN levels in the WT mice, ELISA was performed. We found that with the induction of colitis by using DSS, there was significant decrease in the serum levels of APN. On the contrary, significant increase was found in the serum APN levels of WT mice with exercise as intervention. DSS + EX group showed a significant decrease in the serum adiponectin level (p < 0.03) when compared to Control + EX and showed a significant increase when compared to DSS + SED (p < 0.05). Similar observation were found when compared the serum adiponectin levels of WT mice in Control + SED group with DSS + SED (p < 0.05) and Control + EX (p < 0.01) (Figure 7).

### Activated STAT3 was significantly increased in APNKO mice with acute colitis but significantly decreased by exercise training in APNKO and WT mice with DSS induced acute colitis

**Figure 8:**
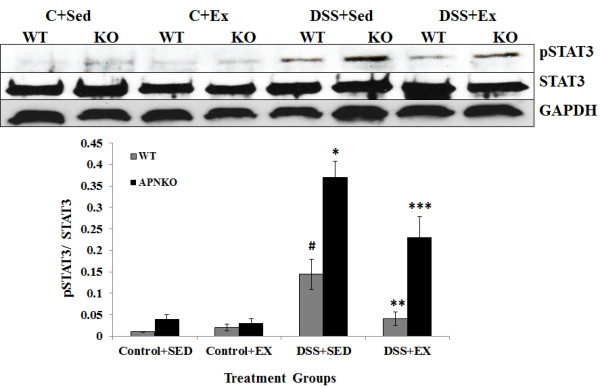
**Western blot analysis of pSTAT3, STAT3 and GAPDH expression.** (A) Representative Western blot image for the expression levels of pSTAT3, STAT3 and GAPDH. (B) Graph showing the ratio of the relative density of pSTAT3 and STAT3 protein expression and normalized with GAPDH. One way ANOVA was used to calculate the significant difference between the relative mean density of pSTAT3 protein expression. # vs * p < 0.04, ** vs *** p < 0.03, # vs ** and * vs *** p < 0.05.

To deduce whether habitual exercise training alters the STAT3 pathway in mice with DSS-induced colitis, STAT3 and pSTAT3 expression in response to DSS treatment in exercise-trained and sedentary APNKO and WT mice was analyzed by Western immuno-blotting. The expression of STAT3 was not significantly altered among the four treatment groups (Figure 8). Exercise was found to significantly decrease the relative intensity of pSTAT3 expression in both the WT (p < 0.05) and APNKO (p < 0.05) mice treated with DSS when compared to DSS + SED group (Figure 8). DSS-treated mice showed significantly higher expression of pSTAT3 (p < 0.01) as compared to the control group except for WT mice in DSS + EX group, which showed no significant difference. In both of the exercise and sedentary groups treated with DSS, pSTAT3 expression was significantly increased in the APNKO (p < 0.01) mice when compared to the WT mice (Figure 8). However, no significance was observed in the control group.

### Epithelial cell proliferation is increased in APNKO mice with acute colitis but unaltered by exercise training in APNKO or WT mice

**Figure 9:**
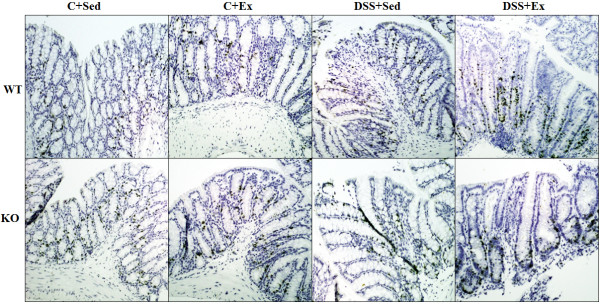
**Effect of exercise training on epithelial cell proliferation.** (A) BrdU positive cells (dark brown) in the mucosa of the transverse colon (magnification = 10×). Representative immunohistochemistry of colonic tissue obtained from control and DSS-treated mice stained for BrdU. All experiments were repeated twice.

**Figure 10:**
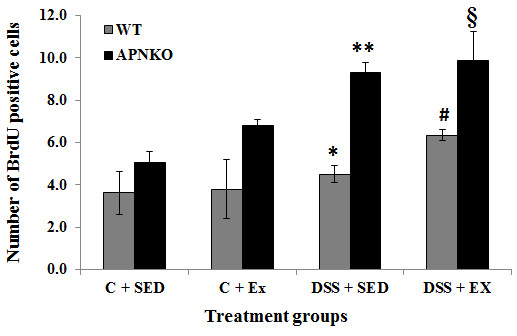
**Effect of exercise training on epithelial cell proliferation as represented by the number of BrdU positive cells counted out of 10 well formed crypts observed in the transverse colon of control and DSS-treated APNKO and WT mice that were either sedentary or exercise-trained.** Two-way ANOVA was used to calculate significant difference between various groups. APNKO C + SED vs ** and * vs ** p < 0.01, APNKO C + EX vs § and # vs § p < 0.05. Values are means ± SE.

In order to determine whether APN deficiency and four weeks of endurance training affect epithelial cell proliferation, sections of the transverse colon from exercise-trained and sedentary APNKO and WT mice were stained for BrdU. Exercise training had no effect on the number of BrdU positive cells in any of the treatment groups. However, epithelial cell proliferation was significantly higher in both the exercise-trained and sedentary APNKO mice that received DSS treatment when compared to their non-treated counterparts (p < 0.05, p < 0.01) respectively. DSS-treated APNKO mice had significantly more BrdU positive cells than the DSS-treated WT mice in both the exercise and sedentary groups (p < 0.05, p < 0.01 respectively), however, epithelial cell proliferation did not differ between the APNKO and WT mice that were not treated with DSS (Figures 9 and 10).

## Discussion

Moderate intensity exercise has been well established to play a protective role either by preventing or by alleviating the symptoms associated with several diseases including obesity, cardiovascular diseases, arthritis, osteoporosis and several others. By the means of this study, we tried to establish the link between the pathological and physical symptoms associated with acute colitis and the effects of moderate intensity exercise and training on C57BL/6 and APNKO mice. DSS induced colitis is a well-established model for acute colitis and it acts by disrupting the epithelium of the colon and results in diarrhea, weight loss, blood in stools and inflammation.

The results of the current study indicate that acute colitis induced by 2% DSS was effective in increasing the clinical score of APNKO mice with SED behavior and WT mice with exercise intervention. Although this difference was insignificant except at day 39, where we observed significantly greater clinical score for the APNKO mice in DSS + SED group when compared to DSS + EX. The clinical scoring was followed by the histo-pathological analysis, which provided greater insight about the degree of inflammation and immune cell infiltration. APNKO mice with sedentary behavior were found to have greater inflammation and infiltration as compared to the exercise group; however no significance was obtained in the WT mice. APNKO mice had greater score than WT mice. These results indicate that APNKO mice are more prone to DSS induce colitis than WT mice [30] and exercise might be play a protective role. This might also point towards faster recovery in the APNKO mice with acute colitis given exercise intervention. This could be explained by the anti-inflammatory effect both APN [30] and moderate intensity exercise by reducing the oxidative tissue damage [27] and increasing the activity of free radical scavengers [31]. However, few contradictory conclusions had been drawn regarding the effects of exercise on ulcerative colitis [32] and hence the effects of exercise remain unclear [33]. The clinical and the histological observations were further supported by the secreted cytokine studies. Significantly elevated levels of both pro-inflammatory cytokines like IL-1β, IL-6 and TNF-α and anti-inflammatory cytokine IL-10 were found in the mice colon with DSS administration except for TNF-α in DSS + EX group. This difference was more significant in the APNKO mice in DSS treated group with sedentary behavior. Despite being an anti-inflammatory cytokine, IL-10 was significantly elevated in all groups with DSS treatment. These results were not unexpected as the increase in IL-10 typically mirrors that of the pro-inflammatory cytokines in an effort to return the intestinal immune system to a state of homeostasis [34,35]. In particular, IL-10 has been implicated in the inhibition of the pro-inflammatory cytokines IL-1β, IL-6, TNF-α and promotes the release of IL-1Ra, which has anti-inflammatory functions of its own [35].

In general, APN has been shown to be pro-inflammatory in IBD through the induction of pro-inflammatory cytokine production and the inhibition of protective growth factors such as IL-6 and macrophage inflammatory protein 2 respectively [6-8]. Furthermore, APN has been shown to exert pro-inflammatory effects in colonic epithelial cells through the induction of various chemokines, including; IL-1β, IL-8, granulocyte-macrophage colony stimulating factor (GM-CSF), and monocyte chemotactic protein-1 (MCP-1) [8]. However, in the current study we found that APN deficient mice were not protected, but more susceptible to inflammation than their WT counterparts as evidenced by significantly greater secretion of IL-6 and TNF-α in the APNKO mice in DSS + EX and DSS + SED groups (Figures 3, 4, 5 and 6), which appears to act via the STAT3 signaling pathway (Figure 8). Our data was similar to the findings by Nishihara et al. [30], who found significantly elevated levels of IL-1β, IL-6 and TNF-α in sedentary APNKO mice treated with 0.5% DSS for 15 days. Previously, in a study conducted at the University of Illinois at Chicago, we used littermates of the APNKO mice (i.e., APN^+/−^ mice) as controls [7]. However, in the current study and that of Nishihara et al. [30], APN^+/+^ (i.e., WT) mice were utilized as controls. Therefore, variability in gut flora (due to the studies being undertaken in different facilities) as well as subtle genetic differences between the WT and APNKO mice are likely in the current study and that by Nishihara et al. ( [2006]), which may possibly account for this discrepancy. However, this requires further confirmation. Moreover, these contradictory results might also be explained through the determination of the predominant isoform of APN present in the colon. Local APN can either be found in a full-length or globular form, and these two molecular isoforms exhibit different binding activity to the APN receptors, and have different biological actions and signal transduction activation [8,36]. Globular adiponectin appears to preferentially act via the AdipoR1 APN receptor [37] and is far more effective at stimulating IL-6, IL-8, IL-10, TNF-α, NFκB, granulocyte-macrophage colony-stimulating factor, and MCP-1 expression than the native form of APN [8,36]. Therefore, future research should determine whether APN isoforms differ in APN^+/+^ and APN^+/−^ mice.

Serum APN levels were significantly increased by exercise intervention and might be one of the factors for the protective role of exercise and reduced secreted cytokine levels in DSS + EX group. However, we didn’t find any significant difference in the intestinal ratio of full length to globular APN in WT mice in all the groups. Also, mice received DSS showed significantly lower level of serum APN when compared to control group. Although, a study conducted in human subject by Weigert et al., 2010 showed that the UC patients have higher serum levels of APN, but there is no mechanistic explanation for this process [38]. However, our study provides many evidences including cytokine profiling, clinical score and pSTAT3 Western blot analysis that points towards the common observation that APNKO mice are more susceptible to UC than WT mice.

Cytokine profiling and serum adiponectin studies were followed by the Western blot analysis for STAT3 phosphorylation. Phosphorylated STAT3 is a well-known marker of inflammation [39]. We observed significantly lower expression of pSTAT3 in the APNKO and WT mice in DSS + EX group when compared to DSS + SED group. Absence of APN was found to have a positive effect on STAT3 activation. These results prove that exercise in combination with APN, might be instrumental in decreasing the status of acute inflammation by reducing the phosphorylation expression of STAT3 in addition to the decrease expression of pro-inflammatory cytokine.

The APNKO mice treated with DSS also displayed greater epithelial cell proliferation (Figure 6) when compared to the DSS-treated WT mice. Since local pro-inflammatory cytokines were elevated in the APNKO mice, it is likely that inflammation-induced damage to the epithelium occurred, necessitating an increase in epithelial cell proliferation to repair the damage [40]. Furthermore, epithelial cell proliferation in response to DSS treatment may have been lower in the WT mice, given that the local APN present in the mucosa of the WT mice may have bound to and inhibited the growth factors HB-EGF and bFGF necessary for regulating colonic epithelial cell proliferation [7].

The mechanism(s) by which exercise affects the gastrointestinal (GI) tract in individuals with IBD is poorly understood, however, decreased blood flow, increased GI motility and permeability, increased mechanical bouncing and increased metabolic stress via neuro-immuno-endocrine alterations have all been suggested to be potentially harmful [5,34,41-43]. Nevertheless, these prospectively harmful effects of exercise have only been investigated following acute bouts of strenuous exercise [5,43,44] and therefore may not be predictive of the long-term consequences of habitual exercise training of a moderate intensity [41]. A major finding of this study was that exercise behavior was not altered in any of the DSS-treated animals, who were able to maintain the prescribed training protocol (without any increased encouragement) for the duration of the study. Furthermore, the clinical symptoms and molecular indicators associated with IBD were alleviated by exercise, suggesting that exercise training may not be harmful and on the contrary may act as a preventive medicine for patients with acute colitis.

Our data also show that exercise training significantly decreased intestinal IL-6 (Figure 3), TNF-α (Figure 6) and IL-1β (Figure 4) (p < 0.05), in the DSS + EX APNKO mice when compared to the DSS + SED APNKO mice. These results suggest that this type of moderate intensity exercise training is sufficient to decrease the local pro-inflammatory response in APNKO mice recovering from acute colitis. However, these cytokine changes may have occurred via a signaling pathway mediated by STAT3, since pSTAT3 expression was also decreased significantly by the exercise training program in both APNKO and WT mice treated with DSS (Figure 8).

Exercise training had no effect on any of the cytokine levels in the DSS + EX WT mice when compared to the DSS + SED WT mice. However, since the sedentary WT mice already appeared to be protected against the DSS-induced inflammatory response, the lack of additional benefits from exercise was not surprising. There is a possibility that water consumption may have been elevated in the exercise-trained WT mice when compared to their sedentary counterparts, thus potentially exposing them to greater quantities of DSS and as a result obscuring any beneficial effects of exercise in the colitic WT animals. Although water consumption was not officially measured, no major discrepancies in water consumption were noted among any of the animals during the experimental procedures; however, this requires clarification. Furthermore, exercise training had no effect on epithelial cell proliferation in either the WT or the APNKO mice (in both the control and DSS-treated groups).

## Conclusions

APN deficiency may act in worsening the symptoms associated with ulcerative colitis by modulating the secretion of local cytokine and epithelial cell proliferation in acute DSS-induced colitis. Moderate intensity exercise could also be beneficial in reducing the symptoms and the secretion of the molecular markers associated with UC. However, it is unknown whether exercise of longer durations (i.e., longer than 4 weeks) will have additional benefits on APNKO mice or whether it will moderate inflammation and symptoms in the WT mice in either a positive or negative fashion. Resistance training could also be used as an intervention to study the effects of exercise in UC and IBD models. More investigation is required to study the pathways and mechanism involved in this process. Moreover, the effect of habitual exercise on chronic colitis remains to be determined. Further research is necessary to clarify whether exercise training is protective or detrimental in mice with acute and chronic colitis and whether the presence of APN will impede or promote the benefits typically associated with exercise training. The determination of the optimal dose of exercise for individuals with both acute and chronic colitis would be of substantial interest to healthcare providers in the use of exercise as a potential adjunct therapy.

## Competing interests

The authors declare that they have no competing interests.

## Authors’ contributions

AS and EF were involved in study concept, design, data acquisition, analysis and interpretation, and writing of the manuscript. BL assisted in animal care. MSB was involved in manuscript review. JLD provided guidance in study concepts, particularly the exercise component of the research design. RF assisted in study design, technical support, data analysis and interpretation and mentorship of the first authors. All authors read and approved the final manuscript.

## Authors’ information

Arpit Saxena and Emma Fletcher have equal first authorship.
